# Olfactory and neurological outcomes of SARS-CoV-2 from acute infection to recovery

**DOI:** 10.3389/falgy.2022.1019274

**Published:** 2022-10-26

**Authors:** Deesha D. Desai, Sophie E. Yu, Brock Salvatore, Zoe Goldberg, Eve M. R. Bowers, John A. Moore, BaDoi Phan, Stella E. Lee

**Affiliations:** ^1^ University of Pittsburgh School of Medicine, Pittsburgh, PA, United States; ^2^ Division of Otolaryngology – Head & Neck Surgery, Brigham and Women’s Hospital, Harvard Medical School, Boston, MA, United States; ^3^ Department of Biological Sciences, University of Pittsburgh, Pittsburgh, PA, United States; ^4^ Department of Otolaryngology – Head & Neck Surgery, Jackson Memorial Hospital, University of Miami, Miami, FL, United States; ^5^ Department of Otolaryngology – Head & Neck Surgery, University of Pittsburgh, Pittsburgh, PA, United States

**Keywords:** SARS-CoV2 (COVID-19), olfaction, cognition, olfactory dysfunction, anosmia, parosmia, hyposmia

## Abstract

**Educational objective:**

To investigate the impact of SARS-CoV-2 on sinonasal quality of life, olfaction, and cognition at different stages of viral infection and evaluate the association between olfaction and cognition in this population cohort.

**Objectives:**

While olfactory dysfunction (OD) is a frequently reported symptom of COVID-19 (98% prevalence), neurocognitive symptoms are becoming more apparent as patients recover from infection. This study aims to address how different stages of infection [active infection (positive PCR test, symptomatic) vs. recovered (7 days post-symptoms)] compared to healthy control patients influence sinonasal quality of life, olfactory function, and cognition.

**Study design:**

Prospective, longitudinal, case-control.

**Methods:**

Participants completed the SNOT-22, University of Pennsylvania Smell Identification Test (UPSIT) and validated cognitive examinations to assess degree of smell loss and neurocognitive function at baseline and at 1 and 3 months for the active group and 3 months for the recovered group. Self-reported olfactory function and overall health metrics were also collected.

**Results:**

The recovered group had the lowest average UPSIT score of 27.6 compared to 32.7 (active) and 32.6 (healthy control). 80% (*n* = 24) of the recovered patients and 56.3% (*n* = 9) of the active patients suffered from smell loss. In follow-up, the active group showed improvement in UPSIT scores while the recovered group scores worsened. In terms of neurocognitive performance, recovered patients had lower processing speed despite an improving UPSIT score.

**Conclusion:**

SARS-CoV-2 infection was found to impact olfactory function in a delayed fashion with significant impact despite recovery from active infection. Although olfactory function improved, decrements in cognitive processing speed were detected in our cohort.

## Introduction

Olfactory dysfunction has often been identified as a hallmark symptom of SARS-CoV-2, presenting in 98% of overall patients, with higher prevalence noted with the wild type variation of the virus (1–4). OD can be quantitative or qualitative, impacting the patient's ability to smell and perceptions of smell. Quantitative OD can range from anosmia (complete loss of olfactory function) to hyposmia (reduced sensitivity) to hyperosmia (oversensitivity). COVID-19 associated OD has several hypothesized mechanisms, including infection of supporting sustentacular cells of the olfactory tract *via* the ACE2 receptor protein and olfactory epithelium damage, with reports of recovery after viral-mediated infection at varying rates (5–7).

Neurocognitive deficits, such as confusion, memory loss, and brain fog, are frequent symptoms of SARS-CoV-2 (8–10). Rates of cognitive decline are significantly higher in SARS-CoV-2 patients compared to control patients, ranging from mild to severe symptoms (11, 12). Several mechanisms have been proposed including local inflammatory changes, axonal injury, production of anti-neuronal autoantibodies, sequelae of hypoxia and coagulopathy, as well as direct viral effects on olfactory, orbitofrontal and brainstem areas (13–15). Recently, SARS-CoV-2 infection has shown to result in reduction in brain size and increased tissue damage in regions connected to the primary olfactory cortex.

Both neurocognitive deficits and OD are complex post-acute sequelae of COVID-19 infection. It is known that olfactory impairment has long been linked to cognitive impairment in several neurodegenerative diseases, with higher prevalence in cohorts with mild cognitive impairment (MCI) compared with control patients (16–20). In adults with MCI, poor odor identification ability has been shown to be predictive of progression to dementia (18, 21, 22).

While reports of anosmia and impaired neurocognitive function during and post COVID-19 infection have become more prevalent, systemic data documenting cognitive performance and the relationship between OD and cognitive performance during active infection and post-recovery is lacking. The current study aims to compare differences in olfaction and neurocognitive function between groups (active COVID-19 patients, recovered COVID-19 patients, and healthy controls) to better understand how SARS-CoV-2 affects sinonasal quality of life, patient perception of smell, smell identification ability, and to assess the relationship between olfaction function and neurocognitive function during and after infection.

## Materials and methods

### Recruitment and eligibility

The study was approved by the Institutional Review Board (IRB) at the University of Pittsburgh as STUDY20040092. All participants provided informed consent prior to participation. Subjects with active COVID-19 infection (active group; positive PCR test, symptomatic), recovered from COVID-19 infection (recovered group; minimum of 7 days post-symptoms), and negative testing for COVID-19 infection (control group) were recruited from testing centers, telemedicine clinic visits, hospitals, social media platforms, and Pitt + Me, a website designed by University of Pittsburgh for research recruitment. 94% of the active group and 97% of the recovered group were ambulatory, home quarantined participants. A REDCap survey, a secure data collection tool, was used to screen participants to determine if they met inclusion criteria. Participant exclusion was determined by a positive response to a past traumatic brain injury, Parkinson's Disease, Alzheimer's Disease, or dementia. Upon passing eligibility, participants were placed into one of the three groups based on the timing and absence/presence of COVID-19 as listed above.

### Pre-assessment

A HIPAA-compliant REDCap database was utilized to collect outcome measures. Participants were first sent a Sinonasal Outcome Test (SNOT-22) to report the severity of their subjective symptoms on a scale from zero (least severe) to five (most severe) and impact on quality of life. Once completed, a health questionnaire was sent to gather each participant's relevant medical history, treatments modalities used to manage symptoms of COVID-19, and symptoms experienced during infection on a Likert scale.

### UPSIT assessment

Following completion of these forms, participants were provided a University of Pennsylvania smell identification test (UPSIT) *via* the postal service (23). Participants self-administered UPSITs remotely due to pandemic constraints. The participants were asked to identify 40 different odors from which a total score was derived to determine the degree of olfactory dysfunction. Each odor was presented to participants as a multiple-choice question, with four different answers available for identification. All UPSIT tests were done remotely, and participants uploaded their multiple-choice answers directly into REDCap. Based on the input into the REDCap database, a cumulative score was generated to determine the degree of olfactory dysfunction. Overall, three modalities, including rhinologic aspects of SNOT-22 testing, UPSIT assessments, and self-reported smell impairment, were used to estimate olfactory dysfunction.

### Neurocognitive assessment

In conjunction with UPSIT, patients also underwent a battery of validated cognitive remote testing through the website CNS Vital Signs (CNSVS) (24, 25). Surveys were pre-selected to assess different components of participants’ neurocognitive functioning through completion of activities specifically targeted to measure important components of cognitive functioning. Exclusion criteria included a history of traumatic brain injury, Parkinson's Disease, Alzheimer's Disease, or dementia. Surveys included: a measure of overall global neurocognitive function [Neurocognitive Index (NCI)], composite memory, verbal memory, visual memory, psychomotor speed, reaction time, complex attention, cognitive flexibility, processing speed, executive function, simple attention, and motor speed. Embedded measures within the software helped evaluate the participant's true testing performance in each category to ensure test validity. These cognitive tests were stratified to provide each participant with a score and percentile based on respective age group. The percentiles provided an index of how each participant scored compared to other subjects of the same age on a scale of 1–99. The higher the percentile, the higher the cognitive measure function.

### Follow-up

Participants in the active group completed these assessments at three study timepoints: during symptomatic COVID-19 (T0), 1 month after initial testing (T1), and 3 months after initial testing (T2). For the recovered COVID-19 group, participants completed this process at baseline and again 3 months after initial testing. The control group completed one baseline evaluation. When providing follow-up responses, participants updated changes in concomitant medications and symptoms from their previous remote study visit.

## Results

The demographics and group distributions are listed in Table 1.

**Table 1:** Demographics and group distributions.

	Active	Recovered	Healthy control
**Sex**			
Female	72% (*n* = 13)	74% (*n* = 23)	59% (*n* = 20)
Male	28% (*n* = 5)	26% (*n* = 8)	38% (*n* = 13)
Transgender male	0% (*n* = 0)	0% (*n* = 0)	3% (*n* = 1)
**Race**			
White	83% (*n* = 15)	76% (*n* = 26)	74% (*n* = 26)
Asian	11% (*n* = 2)	12% (*n* = 4)	11% (*n* = 4)
Hispanic	0% (*n* = 0)	6% (*n* = 2)	9% (*n* = 3)
Black	6% (*n* = 1)	3% (*n* = 1)	0% (*n* = 0)
Native American	0% (*n* = 0)	3% (*n* = 1)	3% (*n* = 1)
Middle Eastern	0% (*n* = 0)	0% (*n* = 0)	3% (*n* = 1)
**Smoking**			
Non-smoker	75% (*n* = 18)	86% (*n* = 31)	79% (*n* = 34)
Smoker	0% (*n* = 0)	3% (*n* = 1)	0% (*n* = 0)
Former smoker	25% (*n* = 6)	11% (*n* = 4)	21% (*n* = 9)
**Age**			
18–26	33.33% (*n* = 6)	32.26% (*n* = 10)	20.59% (*n* = 7)
27–36	27.78% (*n* = 5)	22.58% (*n* = 7)	8.82% (*n* = 3)
37–56	23.22% (*n* = 4)	22.58% (*n* = 7)	23.53% (*n* = 8)
57–66	11.11% (*n* = 2)	19.35% (*n* = 6)	11.76% (*n* = 4)
67–76	5.56% (*n* = 1)	3.23% (*n* = 1)	23.53% (*n* = 8)
77–84	0.00% (*n* = 0)	0.00% (*n* = 0)	11.76% (*n* = 4)
**COVID severity**			
Home quarantine	94% (*n* = 17)	97% (*n* = 30)	N/A
Hospitalized	6% (*n* = 1)	0% (*n* = 0)	N/A
Admitted to ICU	0% (*n* = 0)	3% (*n* = 1)	N/A
**Participant breakdown**			
	18	31	34

### SNOT-22 scores at baseline

The total SNOT-22 score for the active group was 42.3, the recovered group 28.4, and the control group 13.7 (Table 2). Scores from the SNOT-22 subdomains were compared between groups (Table 3). Within the rhinologic domain, the “sense of taste/smell” item in the active and recovered groups were the most greatly impaired for patients. Scores from the psychological dysfunction and sleep dysfunction domains were also compared between groups. The most severe symptom for the active group was “fatigue”, for the recovered group was “waking up tired”, and the healthy controls were distributed between “waking up at night” and a “lack of a good night sleep”. The least severe reported concern was the feeling of embarrassment across all three groups. Based on participant reporting of factors impacting olfactory function and cognition, the active COVID-19 group had the highest SNOT-22 scores in all variables compared to the recovered and control groups.

**Table 2:** Overall SNOT-22 scores.

Group	*n*	Average
Active	18	42.3
Recovered	31	28.4
Healthy control	34	13.7

**Table 3:** Average SNOT-22 (mean) scores by item and COVID group.

	Active COVID-19	Recovered COVID-19	Healthy control
**Rhinologic**			
Need to blow nose	1.8	0.9	0.8
Runny nose	1.6	0.9	0.6
Post nasal discharge drip	1.6	0.8	0.4
Thick nasal discharge	0.9	0.4	0.1
Facial pain pressure	1.3	0.4	0.3
Sense of taste/smell	2.6	3.3	0.5
Blockage/congestion	2.0	1.1	0.6
Rhinologic overall mean	1.7 (se: 0.2)	1.1 (se: 0.1)	0.5 (se: 0.1)
**Extra-rhinologic**			
Dizziness	1.4	0.6	0.3
Difficult falling asleep	1.7	1.4	1.0
Waking up at night	2.0	1.5	1.3
Lack of a good's sleep	2.2	1.9	1.3
Waking up tired	3.1	2.5	1.2
Fatigue during day	3.2	2.3	1.1
Reduced productivity	2.9	1.9	0.9
Reduced concentration	2.8	1.5	0.6
Frustrated/restless	2.3	1.9	0.7
Sad	1.7	1.8	0.4
Embarrassed	1.2	0.6	0.1
Extra-rhinologic overall mean	2.2 (se: 0.3)	1.6 (se: 0.2)	0.8 (se: 0.1)

### Smell-loss impact

#### Self-reported smell impairment between groups at baseline

##### Active COVID-19 infection group

13% (*n* = 2) reported anosmia, 50% (*n* = 8) reported hyposmia, and 38% (*n* = 6) reported no change/normal sense of smell.

##### COVID-19 recovered group

4% (*n* = 1) reported anosmia, 67% (*n* = 18) reported hyposmia, and 30% (*n* = 8) reported no change/normal sense of smell. 6% (*n* = 2) of participants who recovered from COVID-19 reported confusion, less than in the active infection group.

#### Distribution of cognitive percentiles across self-reported smell

There was no detectable correlation between self-reported smell loss, the participant's perspective, and cognitive function within either COVID-19 groups (active or recovered). This linear regression was adjusted for increasing education level and socioeconomic status.

#### UPSIT score assessment across patient groups

Based on UPSIT scores, loss of smell was classified into the following groups, anosmia, hyposmia, or normosmia. The groups’ percentages in each degree of smell loss are reported below (Table 4).

**Table 4:** COVID diagnosis groups’ degree of smell loss in UPSIT testing.

Group	Anosmia	Hyposmia	Normosmia
Active	37.50% (*n* = 6)	18.75% (*n* = 3)	43.75% (*n* = 7)
Recovered	33.33% (*n* = 10)	46.67% (*n* = 14)	20% (*n* = 6)
Healthy control	6% (*n* = 2)	47% (*n* = 16)	47% (*n* = 16)

The active group had a higher percentage of normosmia (43.75%) while the recovered group had a higher percentage of hyposmia (46.67%).

#### UPSIT scores with follow-up

**Figure 1:**
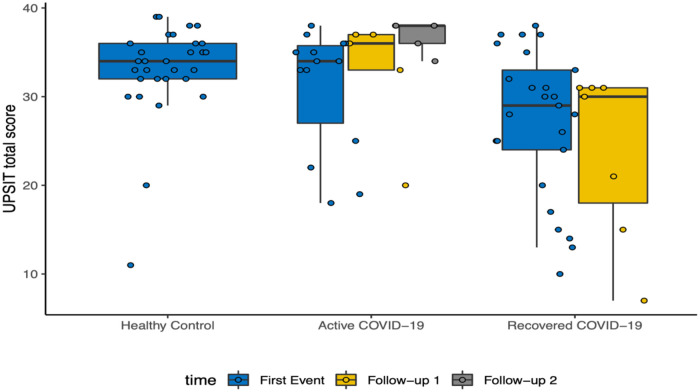
UPSIT scores over the course of follow ups.

The active group had an improvement in UPSIT scores with each follow-up (T1 = 1 month after initial evaluation; T2 = 3 months after initial evaluation). In contrast, the recovered group showed a decline in UPSIT scores at T2 (Figure 1).

### Cognitive percentile analysis

#### Cognitive percentiles across patient groups

Based on the neurocognitive assessment, the distribution of cognitive percentiles across patient groups was analyzed in a linear regression. This was adjusted for increasing education level and increasing income. There was a significant association of lower processing speed in recovered COVID-19 infected patients (*P* value = 0.030). Recovered participants also demonstrated a trend toward lower neurocognitive index scores and diminished psychomotor speed, executive function, and cognitive flexibility (Table 5).

**Table 5:** Subsets of cognitive testing versus COVID diagnosis groups.

	Average test percentile			
Domain	Active COVID-19	Recovered COVID-19	Healthy control	
Cognitive flexibility	48.9	34.8	49.3	
Complex attention	46.6	49.0	59.2	
Composite memory	42.7	43.8	49.7	
Executive function	52.4	34.7	50.1	
Income	1.6	1.8	N/A	
Motor speed	49.1	43.2	52.2	
Neurocognitive index	47.1	35.8	49.2	
Processing speed	57.5	42.0	66.6	
Reaction time	49.0	31.0	36.5	
Simple attention	46.4	49.8	53.6	
Smell loss	0.6	0.9	0.1	
UPSIT group	0.8	1.1	0.6	
Verbal memory	43.1	45.9	47.4	
Visual memory	45.3	45.8	53.3	
**Domain**	**Linear regression test group vs. healthy**			
	**Group**	**Statistic**	***P* value**	**FDR**
Processing speed	Recovered COVID-19	−2.950	0.004	0.030
Neurocognitive index	Recovered COVID-19	−2.009	0.049	0.225
Executive function	Recovered COVID-19	−1.944	0.056	0.226
Psychomotor speed	Recovered COVID-19	−1.807	0.076	0.254
Reaction time	Active COVID-19	1.803	0.076	0.254
Cognitive flexibility	Recovered COVID-19	−1.750	0.085	0.269

#### Distribution of cognitive percentiles with increasing UPSIT score within patient groups

There was a marginal, nonsignificant inverse association (*P* value = 0.122) between UPSIT scores and processing speed in the COVID-19 recovered group; whereas UPSIT scores increased, processing speed decreased. This linear regression was adjusted for education level and socioeconomic status.

#### Cognitive percentiles versus UPSIT total scores

**Figure 2:**
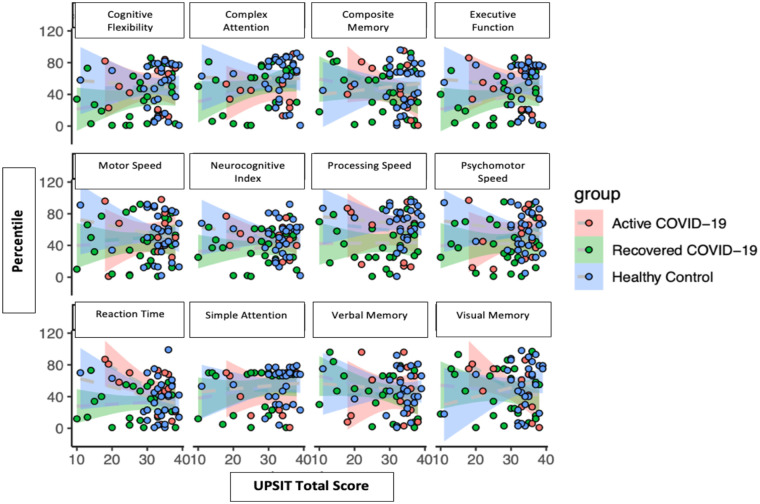
Cognitive domain percentiles to UPSIT scores by groups.

There were no apparent correlations with cognitive percentiles versus overall UPSIT total scores (Figure 2).

#### Cognitive percentiles with follow-up

**Figure 3:**
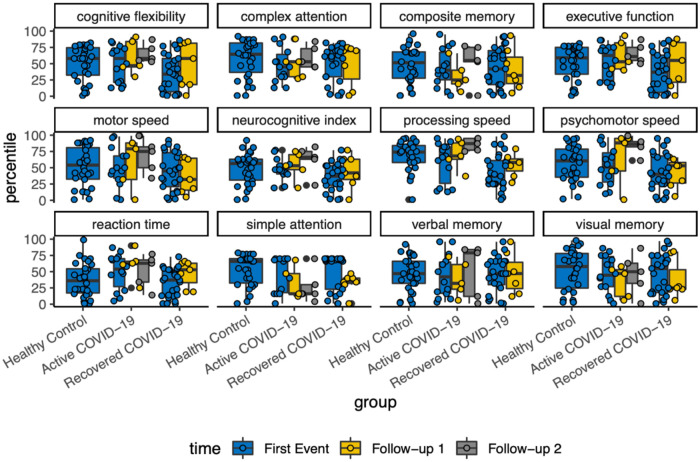
Cognitive percentiles over the course of follow ups.

With each variable of cognitive assessment, there was a scattered distribution among the follow-ups. Overall, there was no detectable changes in neurocognitive function longitudinally (Figure 3).

## Discussion

Although the relationship between COVID-19 infection and olfactory dysfunction has been described, the impact on associated cognitive function has been less clear. Recent work has demonstrated that SARS-CoV-2 viral infection can result in significantly reduced brain size, tissue damage involving the primary olfactory cortex, and damage to orbitofrontal and brainstem regions.

Other viral infections, including MERS-CoV and influenza, have also been associated with neurological decline post-infection (26). It is possible that SARS-CoV-2 may follow a similar trajectory of affecting the CNS and causing persistent effects on cognitive functions following symptomatic recovery from infection (27). Furthermore, a prior study indicated that the hippocampus appears to be vulnerable to coronavirus infections, thus potentially impairing post-infection memory and further cognitive effects (28).

A significant proportion of both active and recovered cohorts reported a major concern with fatigue. This clinical manifestation adds to the phenomenon described as persistent post-acute sequelae of COVID-19 or long-haul COVID in which individuals face symptoms of fatigue, difficulty concentrating, anxiety, among other symptoms after weeks to months following acute COVID-19 infection (29).

Interestingly, in our study, the patients who were considered actively infected had improvement in UPSIT scores over the 4-month study period while the recovered group continued to decline. This may be a result of the potential delay in the manifestation of olfactory dysfunction from initial infection as well as the variability in recovery. These olfactory differences may also be impacted by a risk of selection bias, as participants who opted to partake could have already been experiencing olfactory dysfunction. Of note, more than half of the healthy control group did not experience normosmia at baseline, potentially due to false-negative COVID-19 testing or residual smell loss from a prior COVID-19 infection which was unknown to the participant. Patients also commonly experience distorted smell or “parosmia”, which may be related to disordered regrowth of olfactory epithelia and the time frame of recovery may vary depending on patient factors. Therefore, future studies should plan to focus more closely on the timeline of olfactory dysfunction recovery over a longer time course to discern the variability in olfactory recovery.

Processing speed, the rate at which an individual can recognize and make sense of information, as well as appropriately respond, whether motor, visual, or auditory, allows for a better evaluation of overall cognitive deficit (18). This may be of potential concern for tasks that require greater attention to recognition and call for appropriate, timely responses, such as driving and in certain occupational demands. Furthermore, as seen in other neurodegenerative disorders such as Alzheimer's disease, slowed processing speed may be the first sign of cognitive decline.

Additionally, a finding, though statistically nonsignificant, was a marginal inverse association in which UPSIT scores increased, however, processing speed decreased in the recovered cohort. This finding illustrates that despite improvement in odor identification post-recovery from COVID-19 infection, there may still be long-term neurocognitive deficits, particularly in terms of processing speed.

A recent study has demonstrated a statistically significant relationship between hyposmia and reduced cognitive function in a cohort of 7 COVID-19 patients. This current study did not find a significant association between UPSIT score or patient self-reported olfaction and neurocognitive scores. This could be due to limitations of the UPSIT assessment which is mainly focused on one aspect of smell—assessing odor identification while not providing information about odor discrimination or threshold. It is possible that these other aspects of olfaction may demonstrate a relationship with neurocognitive function that is not revealed in this study. Furthermore, the cognitive assessment utilized by the other study is highly sensitive for the detection of MCI but does not provide the breadth and depth of cognitive assessment provided by the CNSVS survey (30).

This study is not without limitations. First, SNOT-22 has not been validated to study sinonasal quality of life in COVID-19 patients. As a result, the findings may also reflect the impact of systemic antiviral inflammatory response or other associated symptoms of COVID-19. However, the analysis of subdomains as well as cumulative score provides greater insight into patient impairment. Another limitation is the UPSIT assessment, as previously mentioned, does not assess smell discrimination or threshold. UPSIT is regarded as a smell identification especially when compared to other testing modalities such as the CCCRC (smell identification and threshold) and Sniffin’ sticks (smell identification, discrimination, and threshold) (31). This aspect may potentially impact the time of recovery that was recorded through UPSIT testing among the groups. However, given the constraints of the early pandemic, in-person assessments such as the Sniffin’ Sticks test which may have provided more information about qualitative smell loss were difficult to perform. In addition to the UPSIT, we also recorded self-reported smell impairment and rhinologic aspects of the SNOT-22 to supplement the UPSIT data. Of note, the majority of healthy control patients who had any smell disturbance had mild anosmia compared to the active and recovered group. The healthy control group was selected based on negative testing for COVID-19 infection. This baseline olfactory dysfunction may be explained by potential false-negative COVID-19 testing or residual smell loss from a prior COVID-19 infection which was unknown to the participant. There is a risk of selection bias since participants who opted to partake in the study could have already been experiencing olfactory dysfunction. However, all three populations (active, recovered, and controls) were recruited *via* the same platforms, including testing centers, telemedicine clinic visits, hospitals, social media platforms, and Pitt + Me, in an effort to lower this selection bias. Additionally, as this study measured follow-up in participants, it was subject to potential loss of participants over the course of the 4 months. Due to voluntary participation, the study had variability in the number of participants considered among the groups. Our cohort overall was less severe and as a result the findings may not be applicable to the broader population. This study was conducted during the early pandemic period prior to vaccination availability and future studies of interest include exploring parosmia and olfactory dysfunction evaluated through longer-follow-up periods.

In conclusion, SARS-CoV-2 impacts overall sinonasal quality of life and may lead to neurocognitive deficits despite recovery in olfaction. Fatigue was also detected that can persist months after acute infection.

## Data Availability

The original contributions presented in the study are included in the article/Supplementary Material, further inquiries can be directed to the corresponding author/s.

## References

[1] Lechien JR Chiesa-Estomba CM De Siati DR Horoi M Le Bon SD Rodriguez A Olfactory and gustatory dysfunctions as a clinical presentation of mild-to-moderate forms of the coronavirus disease (COVID-19): a multicenter European study. Eur Arch Otorhinolaryngol. (2020) 277(8):2251–61. 10.1007/s00405-020-05965-1 32253535 PMC7134551

[2] Mullol J Alobid I Mariño-Sánchez F Izquierdo-Domínguez A Marin C Klimek L The loss of smell and taste in the COVID-19 outbreak: a tale of many countries. Curr Allergy Asthma Rep. (2020) 20(10):61. 10.1007/s11882-020-00961-1 32748211 PMC7397453

[3] Moein ST Hashemian SM Mansourafshar B Khorram-Tousi A Tabarsi P Doty RL. Smell dysfunction: a biomarker for COVID-19. Int Forum Allergy Rhinol. (2020) 10(8):944–50. 10.1002/alr.22587 32301284 PMC7262123

[4] Hintschich CA Vielsmeier V Bohr C Hagemann J Klimek L. Prevalence of acute olfactory dysfunction differs between variants of SARS-CoV-2-results from chemosensitive testing in wild type, VOC alpha (B.1.1.7) and VOC delta (B.1617.2). Eur Arch Otorhinolaryngol. (2022) 279:1–3. 10.1007/s00405-022-07431-6 35767061 PMC9243797

[5] Brann DH Tsukahara T Weinreb C Lipovsek M Van den Berge K Gong B Non-neuronal expression of SARS-CoV-2 entry genes in the olfactory system suggests mechanisms underlying COVID-19-associated anosmia. Sci Adv. (2020) 6(31). 10.1126/sciadv.abc5801 32937591 PMC10715684

[6] Chen M Shen W Rowan NR Kulaga H Hillel A Ramanathan Jr. M Elevated ACE2 expression in the olfactory neuroepithelium: implications for anosmia and upper respiratory SARS-CoV-2 entry and replication. bioRxiv. (2020) 56(3):. 10.1101/2020.05.08.084996 PMC7439429 32817004

[7] Las Casas Lima MH Cavalcante ALB Leão SC. Pathophysiological relationship between COVID-19 and olfactory dysfunction: a systematic review. Braz J Otorhinolaryngol. (2021) 88:794–802. 10.1016/j.bjorl.2021.04.001 33965353 PMC8068782

[8] Ellul MA Benjamin L Singh B Lant S Michael BD Easton A Neurological associations of COVID-19. Lancet Neurol. (2020) 19(9):767–83. 10.1016/s1474-4422(20)30221-0 32622375 PMC7332267

[9] Carod-Artal FJ. Neurological complications of coronavirus and COVID-19. Rev Neurol. (2020) 70(9):311–22. 10.33588/rn.7009.2020179 32329044

[10] Sampaio Rocha-Filho PA Voss L. Persistent headache and persistent anosmia associated with COVID-19. Headache. (2020) 60(8):1797–9. 10.1111/head.13941 32790179 PMC7436496

[11] Douaud G Lee S Alfaro-Almagro F Arthofer C Wang C McCarthy P SARS-CoV-2 is associated with changes in brain structure in UK biobank. Nature. (2022) 604(7907):697–707. 10.1038/s41586-022-04569-5 35255491 PMC9046077

[12] Liu Y-H Chen Y Wang Q-H Wang L-R Jiang L Yang Y One-year trajectory of cognitive changes in older survivors of COVID-19 in Wuhan, China: a longitudinal cohort study. JAMA Neurol. (2022) 79(5):509–17. 10.1001/jamaneurol.2022.0461 35258587 PMC8905512

[13] Koyuncu OO Hogue IB Enquist LW. Virus infections in the nervous system. Cell Host Microbe. (2013) 13(4):379–93. 10.1016/j.chom.2013.03.010 23601101 PMC3647473

[14] Mukaetova-Ladinska EB Kronenberg G Raha-Chowdhury R. COVID-19 and neurocognitive disorders. Curr Opin Psychiatry. (2021) 34(2):149–56. 10.1097/yco.0000000000000687 33395101 PMC7924920

[15] Hosp JA Dressing A Blazhenets G Bormann T Rau A Schwabenland M Cognitive impairment and altered cerebral glucose metabolism in the subacute stage of COVID-19. Brain. (2021) 144(4):1263–76. 10.1093/brain/awab009 33822001 PMC8083602

[16] Manca R De Marco M Ince PG Venneri A. Heterogeneity in regional damage detected by neuroimaging and neuropathological studies in older adults with COVID-19: a cognitive-neuroscience systematic review to inform the long-term impact of the virus on neurocognitive trajectories. Front Aging Neurosci. (2021) 13:646908. 10.3389/fnagi.2021.646908 34149394 PMC8209297

[17] Eibenstein A Fioretti AB Simaskou MN Sucapane P Mearelli S Mina C Olfactory screening test in mild cognitive impairment. Neurol Sci. (2005) 26(3):156–60. 10.1007/s10072-005-0453-2 16086128

[18] Djordjevic J Jones-Gotman M De Sousa K Chertkow H. Olfaction in patients with mild cognitive impairment and Alzheimer's disease. Neurobiol Aging. (2008) 29(5):693–706. 10.1016/j.neurobiolaging.2006.11.014 17207898

[19] Churnin I Qazi J Fermin CR Wilson JH Payne SC Mattos JL. Association between olfactory and gustatory dysfunction and cognition in older adults. Am J Rhinol Allergy. (2019) 33(2):170–7. 10.1177/1945892418824451 30632380

[20] Dintica CS Marseglia A Rizzuto D Wang R Seubert J Arfanakis K Impaired olfaction is associated with cognitive decline and neurodegeneration in the brain. Neurology. (2019) 92(7):e700–9. 10.1212/wnl.0000000000006919 30651382 PMC6382360

[21] Devanand DP Michaels-Marston KS Liu X Pelton GH Padilla M Marder K Olfactory deficits in patients with mild cognitive impairment predict Alzheimer's disease at follow-up. Am J Psychiatry. (2000) 157(9):1399–405. 10.1176/appi.ajp.157.9.1399 10964854

[22] Fusetti M Fioretti AB Silvagni F Simaskou M Sucapane P Necozione S Smell and preclinical Alzheimer disease: study of 29 patients with amnesic mild cognitive impairment. J Otolaryngol Head Neck Surg. (2010) 39(2):175–81. 10.2310/7070.2009.090046 20211105

[23] Doty RL Shaman P Kimmelman CP Dann MS. University of Pennsylvania smell identification test: a rapid quantitative olfactory function test for the clinic. Laryngoscope. (1984) 94(2 Pt 1):176–8. 10.1288/00005537-198402000-00004 6694486

[24] Gualtieri CT Johnson LG. Reliability and validity of a computerized neurocognitive test battery, CNS vital signs. Arch Clin Neuropsychol. (2006) 21(7):623–43. 10.1016/j.acn.2006.05.007. 17014981

[25] Signs CV. CNS Vital Signs Interpretation Guide. CNS Vital Signs, LLC (2021).

[26] Bohmwald K Gálvez NMS Ríos M Kalergis AM. Neurologic alterations due to respiratory virus infections. Front Cell Neurosci. (2018) 12:386. 10.3389/fncel.2018.00386 30416428 PMC6212673

[27] Montalvan V Lee J Bueso T De Toledo J Rivas K. Neurological manifestations of COVID-19 and other coronavirus infections: a systematic review. Clin Neurol Neurosurg. (2020) 194:105921. 10.1016/j.clineuro.2020.105921 32422545 PMC7227498

[28] Ritchie K Chan D Watermeyer T. The cognitive consequences of the COVID-19 epidemic: collateral damage? Brain Commun. (2020) 2(2):fcaa069. 10.1093/braincomms/fcaa069 33074266 PMC7314157

[29] Groff D Sun A Ssentongo AE Ba DM Parsons N Poudel GR Short-term and long-term rates of postacute sequelae of SARS-CoV-2 infection: a systematic review. JAMA Netw Open. (2021) 4(10):e2128568. 10.1001/jamanetworkopen.2021.28568 34643720 PMC8515212

[30] Pirker-Kees A Platho-Elwischger K Hafner S Redlich K Baumgartner C. Hyposmia is associated with reduced cognitive function in COVID-19: first preliminary results. Dement Geriatr Cogn Disord. (2021) 50(1):68–73. 10.1159/000515575 33853062 PMC8089429

[31] Trecca EMC Cassano M Longo F Petrone P Miani C Hummel T Results from psychophysical tests of smell and taste during the course of SARS-CoV-2 infection: a review. Acta Otorhinolaryngol Ital. (2022) 42(Suppl. 1):S20–35. 10.14639/0392-100X-suppl.1-42-2022-03 35763272 PMC9137382

